# Sex Differences in Brain Injury and Repair in Newborn Infants: Clinical Evidence and Biological Mechanisms

**DOI:** 10.3389/fped.2019.00211

**Published:** 2019-06-26

**Authors:** Ted S. Rosenkrantz, Zeenat Hussain, Roslyn Holly Fitch

**Affiliations:** ^1^Division of Neonatology, Department of Pediatrics, University of Connecticut School of Medicine, Farmington, CT, United States; ^2^Department of Volunteer Services, UCONN Health, Farmington, CT, United States; ^3^Department of Anthropology, New York University, New York, NY, United States; ^4^Department of Psychology, University of Connecticut, Storrs, CT, United States

**Keywords:** sex, gender, infant, newborn, neurological brain, injury, repair

## Abstract

Differences in the development of the male and female brain are an evolving area of investigation. We are beginning to understand the underpinnings of male and female advantages due to differences in brain development as well as the consequences following hypoxic-ischemic brain injury in the newborn. The two main factors that appear to affect outcomes are gestation age at the time of injury and sex of the subject. This review starts with a summary of differences in the anatomy and physiology of the developing male and female brain. This is followed by a review of the major factors responsible for the observed differences in the face of normal development and hypoxic injury. The last section reviews the response of male and female subjects to various neuroprotective strategies that are currently being used and where there is a need for additional information for more precise therapy based on the sex of the infant.

## Introduction

Care and management of sick and premature newborn infants is generally based on specific diagnoses and conditions and does not take into consideration the infant's sex. Infant sex can play a major role in disease onset, course and resolution. A well-known example is respiratory distress syndrome or RDS. It is well-documented that surfactant production in the fetal lung is sex dependent (1). It is confirmed by the lower incidence of RDS in females than males (2). Even in the era of improved therapy for RDS that includes antenatal corticosteroids and postnatal treatment with surfactant therapy, the gender gap in respiratory outcomes has not narrowed. Additionally the utilization of these therapies have not changed for male and female preterm infants despite our current knowledge on the influence of the fetal/infant's sex.

It likely that the lung is not the only organ system that functions differently in males and females. In a study aimed at investigating the effect of sex on survival and short-term outcomes of very low birth weight infants (VLBWIs) born in Japan (2003 and 2012- Neonatal Research Network of Japan) with the primary outcome of a composite of mortality or any major morbidity, including neurologic injury, bronchopulmonary dysplasia (BPD), necrotizing enterocolitis (NEC), or retinopathy of prematurity requiring treatment, it was shown that the composite primary outcome was worse in males (3). A recent report from the Vermont Oxford Network NICUs reiterates the outcome differences between the sexes in premature infants (4).

Differences in behavioral and psychological responses between the sexes are well-known. Only recently has there been documentation for this finding including fundamental cellular, molecular and physiological differences, especially as they pertain to neuronal function, brain injury and the response to the injury, and to therapy. In fact recent data suggests that therapies may need to be sex-specific to have maximal effectiveness and best outcome (5).

The biological underpinnings of sexual differentiation in males and females have traditionally been considered to be based on sex chromosomes and production of sex hormones that interact with their cellular receptors. Many conditions in human health and disease are sexually dimorphic with different manifestations and outcomes in males and females. More recently it has become apparent that these differences exist independent of hormonal differences and that the differences are not just in the input of signal through the receptor to the cell but there are differences in the cellular output and response. The anatomic and pathologic correlates of this have been well-described with many clinical conditions, including cerebral ischemia and stroke, in the developing male and female brain (6, 7). Given the evidence that male and female infants have different neurodevelopmental profiles it is important to examine the evidence for the cellular, molecular, hormonal, and/or physiological reasons for these differences. Our research group has done studies utilizing the Rice Vannucci model of hypoxic ischemic injury in preterm and term equivalent rat pups and evaluated cellular, histological, physiological, and behavioral outcomes based on sex (8–12). We have found that despite similar histological findings after brain injury, there were significant differences in various domains of neuro-behavioral responses and outcomes based on sex.

The focus of this review is to explore what is currently known about the influence of sex on fetal and neonatal brain development, injury, therapy, and outcome. Currently fetal, neonatal, and post-neonatal care pertaining to the brain and neurological conditions does not take into consideration the sex of the fetus or infant. Thus, there is no attention to sex specific management for neonatal conditions such as intraventricular hemorrhage, periventricular leukomalacia, perinatal infection, inflammation associated with chorioamnionitis, and diffuse perinatal cerebral hypoxic-ischemic events. The importance of neonatal sex dimorphism as part of treatment strategies in infants is best illustrated by the studies on prophylactic indomethacin for prevention of intraventricular hemorrhage (IVH) in premature infants in the perinatal period. An initial RCT by Ment et al. showed that prophylactic indomethacin given to very low birth premature infants soon after birth helped in reducing the incidence and severity of IVH (13). This finding was followed by wide-spread use of prophylactic indomethacin in this population. When data were re-analyzed several years later, entering male sex as a primary variable, it was discovered that only the male infants had benefited from prophylactic indomethacin (14). Had there been sex based evaluation of outcomes in the initial study, the use of this therapy could have been more specifically applied.

Currently magnesium sulfate is given to women believed to be at risk for delivering a preterm infant. The therapy is given specifically to prevent cerebral palsy. Caffeine is given to preterm infants to decrease the incidence of BPD and cognitive impairment. Term infants who have evidence of hypoxic ischemic encephalopathy are treated with hypothermia. None of the trials that led to our current use of these therapies have taken into account the sex of the infant.

This paper will focus on what is known about the sex differences in brain structure and function, cellular, and molecular mechanisms underlying such differences and their variable responses to therapies following hypoxic ischemic injury. Such knowledge should lead to sex specific therapies following brain injury.

### Gross Structural and Functional Differences in the Male and Female Nervous Systems

In reviewing sex differences it is important to first examine the anatomy and physiology of the normal brain prior to examining issues of brain injury and therapy.

#### Neuro-Anatomical and Hormonal Differences Between Sexes

There are many sexually dimorphic regions in the brain, generally as a result of hormonally influenced cell growth and apoptosis during brain development. Gonadal androgens are critical for the development and maintenance of sexually dimorphic regions of the male brain that are responsible for male-specific behavior and physiological functioning (15). Different expression of estrogen and testosterone receptors on the neuron in the different sexes may also play a role (16). The hormonal control of cell death is currently the best-studied mechanism for explaining sex differences in cell number in the brain and spinal cord. For example, males have more cells than do females in the principal Bed Nucleus of the Stria Terminalis (BNSTp) and in the Spinal Nucleus of the Bulbocavernosus (SNB), whereas females have a cell number advantage in the AnteroVentral PeriVentricular nucleus (AVPV). In each case, the difference in cell number by adulthood correlates with a sex difference in the number of dying cells at some point in development (17). The difference in apoptosis between males and females appears to be responsible for sexual differentiation and lateralization of neuronal number in a discrete forebrain nucleus (SDApc) which influences masculine vocal emission in the gerbil (18). In animal studies the medial PreOptic Area (mPOA) of the hypothalamus is found to contain a Sexually Dimorphic Nucleus (SDN-POA) that is 5–7 times larger in males than females and plays a part in the development and expression of male-specific sex behaviors in adulthood. These differences in the size of the SDN-POA are thought to be related to estrogen-associated effects on apoptosis during early development. Aside from a critical role for estrogen, the mechanisms that establish and maintain this sex difference are largely unknown (19).

Male rats have more neurons than females in the primary visual cortex, with 19% more neurons than female rats in the binocular region and 18% more in the monocular region of the primary visual cortex (20, 21). In other animals such as Xenopus laevis, where the laryngeal motor nucleus (nucleus of cranial nerves IX-X) is part of a sexually differentiated, androgen-sensitive neuromuscular mechanism devoted to vocalization, males have more IX-X neurons than females (22). For all of the sex related differences it is not clear to what extent sex hormones, receptors, and male/female function drive these dimorphic differences and what is due to other factors.

Role of sex hormone effects in the sexually dimorphic areas of the brain such as the medial Pre-Optic Area (mPOA) of the hypothalamus and the Spinal Nucleus of the Bulbocavernosus (SNB) in the rat are modulated differently in males and females and this effect is mediated by alterations in cell preservation and cell death (23, 24). The action of testosterone as well the aromatization of gonadal derived testosterone to estradiol in the brain play important roles in developing this sexual dimorphism (19, 25).

In studies of the rat Spinal Nucleus of the Bulbocavernous (SNB), the interaction of brain-derived neurotrophic factor (BNDF) with the androgen receptor (AR), and the activation of androgen-response elements (ARE) on the gene appear to be closely interconnected (15). A perinatal surge in androgen activity in male rat fetuses not only helps in preventing programmed cell death of the SNB in males but also preserves the muscles that this nerve innervates (26). In male songbirds the sexually dimorphic nuclear complex involving high vocal control nuclei (HVC), and Robust Arcopallium (RA) nuclei appear to be regulated not just by the direct effect of androgens on cell preservation but also by estrogen receptor-containing cells that create dimorphisms in cellular specification (27, 28).

Hormonal modulation of apoptotic process has been well-documented especially during fetal and neonatal brain development (23). There is a greater and more prolonged apoptosis in females in selected areas of the developing brain resulting in female rats with a smaller visual cortex than males and female gerbils with a lesser number of neurons in the primary forebrain nucleus responsible for vocalization (18, 21, 29). The mechanism by which androgens and estrogens regulate cell number in sexually dimorphic regions of the brain is most likely linked to their influence on cell death and apoptosis rather by their influence on neurogeneisis. It has been shown that sex hormones influence this mechanism by their effect on nuclear Androgen Receptors (AR) or Estrogen Receptors (ER) which work via effectors such as bax and bcl2 proteins that regulate apoptosis (17). Pathways downstream from hormone and receptors may also show sexual dimorphism. For example, the handling of Ca related cellular signaling in neurons is sexually dimorphic and but may also be related to the effects of androgens in the first week of life (30).

As noted above it is not clear if the anatomical difference is due to growth or apoptosis and the role of sex hormones (30). It is clear that fetal brain exposure to male and female hormones has lasting effects on brain development and subsequent behavior. The male hippocampus, responsible for complex cognitive and emotional responses, is larger than that of the female but it is unknown if this is due to increased growth or less cell death and what is the purpose it serves. Male neonatal hippocampal cells appear to be protected from glutamate induced cell death in the presence of DHT whereas the female cells suffer significant injury. Bowers et al. explored the influence of estradiol on hippocampal cells (31). Male and female rat pups were injected with estradiol at birth. The hormone only affected females with increase proliferation of neurons. Estrogen antagonists decreased cell number only in the males. It was noted that the increased cell number in the female was due to increase proliferation and not decreased cell death. It also appears that the basal cell production in the male must require some critical amount of estrogen for normal cell production and hippocampal development (31).

#### Neuro-Physiological Differences

Observations from clinical research in humans have suggested a difference in brain and neuronal physiology based on sex differences starting in the fetal and newborn period and extending through the human lifespan into adulthood. In a retrospective review of human case fatality data following traumatic brain injury, males had significantly more mortality for similar injury severity suggesting a difference in injury response and physiological repair mechanisms (32). Similarly, cerebral palsy and related disorders of neurological injury during perinatal development are more common in males (33). Not only are male preterm infants more vulnerable to intraventricular hemorrhage and white matter injury compared to similar gestational age females, but their repair processes, healing and ultimate neurodevelopmental outcomes also appear to be worse (33). Physiological regulation such as cerebral blood flow regulation also varies with sex but the differences vary with age. In premature infants, girls have significantly lower CBF than boys of similar gestational and postnatal age (34); however, adults females have higher cerebral blood flow than males (35). The mechanism regulating this are not well-understood but the relative immaturity of cerebral blood flow auto regulation in premature infants may be the reason why females with a relatively lower cerebral blood flow have lesser incidence of germinal matrix or intraventricular hemorrhage.

Further investigation into the mechanism of many of the above clinical observations appear to indicate basic physiologic and pathophysiologic differences in apoptotic pathways following hypoxic-ischemic injury. In animal experiments, it has been demonstrated that the apoptotic pathway in males with hypoxic neuronal injury tends to involve PARP-1 and apoptosis inducing factor (AIF). Females, on the contrary, have involvement of cytochrome c-caspase 3 pathways for apoptosis (36). Similar results were found in *in-vitro* experiments without external influences such as sex-specific hormones, suggesting that the sex differences may be due to differences in intrinsic genetic programming and subsequent physiology (33, 36, 37).

The location in brain where external modifiers act may also be sexually dimorphic. Early life stress and neuropathic pain are known to affect neurological responses to later life events in humans, but the effects of the same stressor may involve different brain locations in males and females. Phosphorylated Extracellular signal-Regulated Kinase (p-ERK) activation has been used to study neuronal response to stress. In animal studies using this marker it was found that early life stress induced by maternal separation and social isolation (MSSI) increased p-ERK in the paraventricular nucleus (PVN) and amygdala of male mice but the response in female mice was in a different part of the brain, *viz*. in the medial prefrontal cortex and nucleus accumbens. However, combination of MSSI and neuropathic pain increased p-ERK in the PVN and amygdala of female mice (38). In another study evaluating the difference in pain responses, the effect of vagal stimulation or ablation on neuropathic pain and its response to opioids for tempo-mandibular joint disease was demonstrated to be more severe in female than in male rats. This finding correlates well with the clinical finding of higher prevalence of tempo-mandibular pain in women (39).

From these observations and experimental findings it seems very likely that not only are there anatomical and hormonal differences that account for sex differences in neurologic response to adverse events but there may be intrinsic cellular pathways that are different in males and females. A more detailed evaluation of such molecular, metabolic, and cellular responses is important.

### Differences in Cellular and Molecular Mechanisms Between Males and Females

Apart from obvious anatomical and physiological changes between males and females of various species of animals, there is emerging evidence of changes in fundamental cellular and molecular differences between the sexes. Processes of cell proliferation, cell death with differences in cytosolic and mitochondrial mechanisms have been shown to have sexual dimorphism.

#### Apoptosis and Cell Death

**Figure 1:**
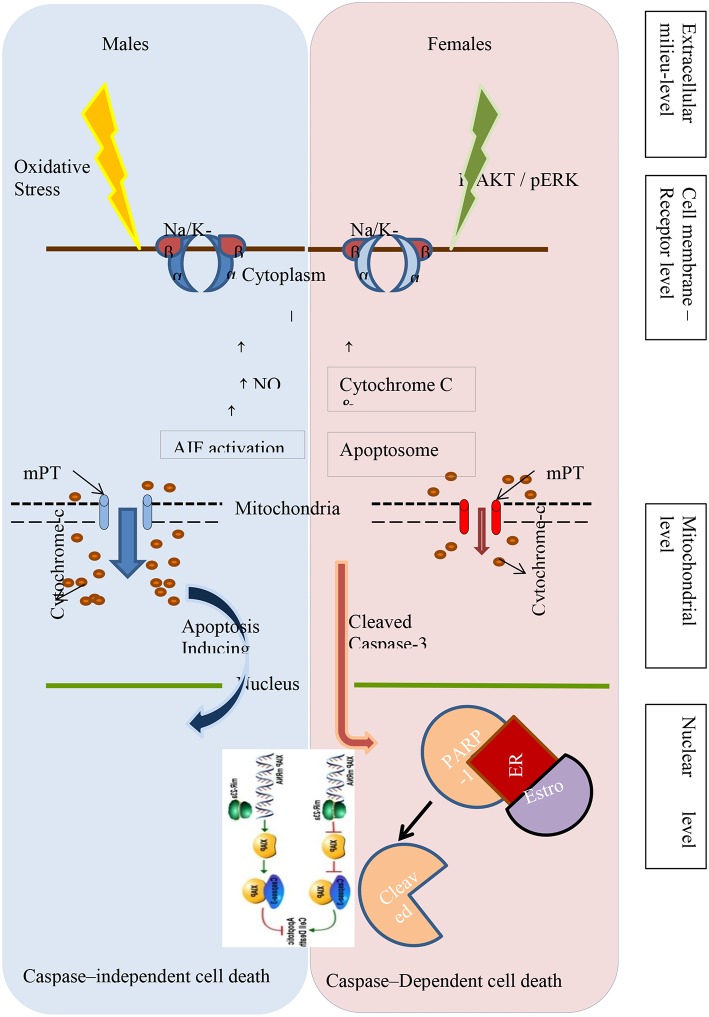
Different pathways of programmed cell death in the male and female newborn brain following hypoxic-ischemic injury.

While investigating the underlying mechanisms of sexual dimorphism in neuronal cell death during normal brain development, it is becoming increasingly clear that the fundamental pathways of programmed cell death are different in males and females of the species. While studying postnatal development of lateral septum in mice brain, there is evidence that apoptosis involving changes in both single-stranded DNA and caspase-3 are different in males and females (40). More evidence of differences between the sexes in caspase-dependent and caspase-independent programmed cell death comes from studies of neonatal hypoxic ischemic injury in animals. A summary of the various cellular mechanisms that differ between males and females are shown in Figure 1.

- **Cell Glutathione levels:** In a series of experiments conducted using developing neurons derived separately from male (XY) and female (XX) rats, Du et al. have delineated the innate differences in cell death pathways (41). Male cells are more susceptible to nitrosative stress and glutamate related excitotoxicity with triggering of Apoptosis Inducible Factor (AIF) and caspase independent cell death or necrosis. On the contrary, when female cells were exposed to a cytotoxic agent such as etopside and sturosporine they were able to trigger a caspase-3 mediated programmed cell-death response mediated by cytochrome-c. They explained this by the innate difference in cellular glutathione anti-oxidant effects where female cells were able to maintain a higher glutathione levels under stress, irrespective of effect of sex hormones (41).
- **Caspases:** Caspase activation dependent apoptosis has consistently been found to be the dominant mechanism of apoptosis in females (42). After hypoxic ischemic (HI) injury, it has been shown that caspase 3 and 7 levels are 3–6-fold higher in female cortex and hippocampi. In other experiments using the Rice-Vannucci model of hypoxic ischemic injury it was demonstrated that not only was caspase activation responsible for cell death selectively in females but also they were less susceptible to oxidative damage and the effects of treatment were also sexually dimorphic (43). Sex -differences in caspase 8 expression along with antiapoptotic protein bcl-2 have also been incriminated in the increased susceptibility of male rats to servoflane anesthesia related brain injury and subsequent cognitive deficits (44). In any case, if females do follow a caspase pathway to apoptosis after HI injury, it would explain why females may derive greater benefit from therapeutic hypothermia as opposed to males as the caspase pathway is very temperature dependent. It would also suggest that other interventions may be needed to preserve neurons in the male brain in the clinical setting.
- **Apoptosis Inducing Factor (AIF):** Just as caspase related mechanisms are dominant in females, Poly-ADP-Ribose-Polymerase (PARP) and AIF mechanisms causing apoptosis are dominant in males. AIF which translocates from mitochondria to nucleus for initiation of apoptosis was more prominent in male immature animal brains after injury. Under similar conditions, in females, neuronal caspase-3 showed more prominent activation. However, the role of nitrotyrosine formation and autophagy were not different between sexes (45). Poly(ADP-ribose) polymerase (PARP), a ubiquitous nuclear enzyme is activated by various injuries and using NAD helps in synthesizing Poly (ADP-ribose) or PAR and thus helps to stabilize and repair DNA. Its isoform PARP-1 is the most active and important. Increased PARP activity however can deplete NAD leading to lack of this substrate for mitochondrial function of ATP production (46, 47). In males, PARP activation initiates the cascade of events leading to depletion of NAD causing mitochondrial energy failure and resulting in cell death. However, in females, with similar PARP activation, NAD depletion does not occur. These molecular mechanisms may be fundamental to the sex differences in brain injury and repair (46, 47).
- **X-linked inhibitor of apoptosis (XIAP):** Another reason for female cells preservation may be the presence of the X-linked Inhibitor of Apoptosis factor in humans which directly inhibits at least two members of the caspase family of cell-death proteases, caspase-3 and caspase-7 (48, 49). Animal experiments in a stroke model of brain injury using microRNA-23A to block the translation of XIAP was shown to decrease XAP-caspase 3 association resulting in an increase in available caspase 3 and promotion of apoptotic cell death. This may explain why, and adds to the evidence that, ischemic neuronal death in females occurs predominantly by caspase-dependent pathways (6). It has also been shown that males and females have varying processes for XIAP effects based not only on intrinsic gene expression but also the external sex hormonal mileu (50). Regulation of XIAP via miRNA 23A and a plant derived small molecule, embelin, are being considered as therapeutic targets for future research although the effect of XIAP is still speculative and direct evidence of protection rendered by this factor is still under investigation (51–53).
- **Other Cellular Factors:** Apart from the above listed factors, other differences have also been noted in the apoptotic mechanism in males vs. females. The pro-apoptotic molecule p53 has been shown to have sexually dimorphic effects. In UV induced apoptosis in brain Sub Ventricular Zone (SVZ) cells, estrogen offered protection via its effects on p53 but testosterone did not have this effect (54). Apoptosis differences between sexes may also be influenced by sexual differences in COX-2 expression. In rat models of traumatic brain injury it has been shown that increase in COX-2 expression in male rats correlates with increased apoptotic cell death but not with neuronal necrosis (55). There may also be other parts of apoptotic-antiapoptotic pathways such as Bax, bcl-2 etc., that may be different among the sexes. *BAX*—a pro-apoptotic gene and *bcl-2* family of genes that are anti-apoptotic may have different expression in males and females and in different areas of the brain. Their effect may be direct or indirect through their regulation by estrogens and testosterone (17, 56). Apart from cell death related pathways and modulators, cell proliferation, and proliferating progenitor cells may also show sexual dimorphism, but more evidence is awaited (57).

#### Mitochondrial Differences in Males and Females

Mitochondrial dysfunction and subsequent energy metabolism deficiencies are important in mediating cell function and cell death. Testing the brain effects of male and female rats with induced hypoxia ischemia suggests that mitochondrial dysfunction in the HI-induced brain may be sexually dimorphic (58). Following HI, male rats were observed to be more susceptible to brain mitochondrial dysfunction than female rats and this was related to increase in mitochondrial proteins present in the electron transport chain complexes I, II, and IV in female brains and not in male brains (58, 59). Other clinical and experimental studies exploring this phenomenon suggest that lower basal glutathione levels, lower post-hypoxic mitochondrial glutathione peroxidase (mtGPx) activity, and mitochondrial glutathione peroxidase 4 (mtGPx4) protein levels contribute to the relative susceptibility of male brain damage by oxidative stress and mitochondrial dysfunction (60). In contrast, female subjects showed more resilience against mitochondrial dysfunction following hypoxia-ischemia due to increased stimulation of electron transport chain proteins. Experiments in 8-day old rats subjected to HI and treated with acetyl-L-carnitine found that citrate synthase activity was increased in the ipsilateral hemisphere of both male and female rats (60). However, levels of mitochondrial protein complexes I, II, and IV only increased in the female brain (60). In addition, the mitochondrial biogenesis-associated transcription factor NRF-2/GABP-alpha increased in females but decreased in males (59). This suggests that one of the reasons for different susceptibility to neurologic injury may be the differences in mitochondrial related pathways in male and female cells with female cells showing more resilience to injury.

#### Inflammatory Biomarker Differences in Males and Females

Inflammatory mediators such as chemokines, cytokines, and other biomolecules play an important role in brain cell injury following HI in the newborn. Savman et al. studied pro- and anti-inflammatory cytokines in infants who met criteria of birth asphyxia and whose encephalopathy was staged with the criteria of Sarnat (61). There was a strong correlation between IL-6 and 8 and the degree of encephalopathy while there was no correlation with TNF or any of the other cytokines tested (61). Using the Rice-Vannucci model in term-equivalent newborn mice, Mirza et al. examined the timing of inflammatory response (62). At 24-h post HI, there were no differences in IL-1β and TNF but at 3 days, males had significantly higher levels than the females. There was greater neutrophil and lymphocyte infiltration into the brain at day 3 in the males vs. females while there initially was no difference on day 1. Infarct size correlated with the elevated cytokine levels in the males. On follow-up after injury, females exhibited fewer behavioral abnormalities. Interestingly, at this young age, there were no differences in testosterone or estradiol between the males and females at day 1 or day 3 (62).

Microglial activation, which plays an important role in brain repair and neuro-inflammatory response to injury may also be sexually dimorphic with males having microglia primed for activation in excess to that in females following HIE in the rat (62). There was also a similar sex difference seen in the peripheral inflammatory response with higher serum levels of IL-1β and TNF-α and infiltration of peripheral leukocytes seen in males (62).

In our own lab we have demonstrated specific behavioral abnormalities in pre-term and term rats that varied by sex (63). Using our P6 HI rat model, we found that late or “chronic” inflammatory factors such as Rantes (chemotactic cytokine ligand 5; CCL5), MCP-1 (CCL2), and MIP-1a (CCL4) were all significantly elevated in serum at 48 h in HI pups, and these levels were highly predictive of subsequent behavioral and neuropathologic outcomes measured months later. Based on our success using caffeine as a neuroprotectant in this population, we are currently studying its administration in a group of preterm rat pups to determine its effects on the inflammatory biomarkers by sex (64).

#### Neurosteroid Differences in Males and Females

As reviewed above, endogenous sex hormonal influences in the brain and nervous system of the fetus and newborn infant have been well-documented but the effect of local brain steroids or neurosteroids is just emerging. Neurosteroids in the developing fetal brain are important in protective and neurodevelopmental processes and modulation of these hormones may be useful in future therapeutic fetal neuroprotective strategies.

Testosterone and its metabolites function as neurosteroids and have local effect on the fetal and neonatal brain. However, the results of studies of testosterone and brain injury are inconsistent. In studies with postnatal rats subjected to HI, injured males and testosterone treated females had worse pathological changes and neurodevelopmental outcomes than sham treated females (8).

The role of estrogens in brain recovery after HI was explored in a rat model focusing especially on hippocampal neurogenesis and it was shown that the beneficial effects seen in females were not due to neurogenesis or death but due to other components of neural responses (65).

The interaction of estrogen and testosterone in brains of infants is complex because testosterone is a precursor of estradiol via the action of aromatase enzyme which has high activity in the brain. Therefore, higher local brain testosterone may result in relatively high estradiol as well. However, administration of estradiol in animal models of hypoxic ischemia had shown improved long term outcomes. The effect is sex specific with evidence of increased short-term cell genesis in hippocampus of females (65).

In excitotoxic GABA_A_ mediated insult using Muscimol in a rat model, it was demonstrated that both the sex-related hormonal milieu and the developmental stage of the brain had an effect on the extent and sex-specificity of the injury (66). The fetal male rat was more susceptible than the adult male (66).

Based on the findings of better brain recovery from injury in females, the hypothesis was tested in a pilot clinical trial (ProTECT trial) that progesterone therapy after traumatic brain injury will improve recovery in males (67). The results were promising with no evidence of any adverse effects. However, larger phase III trials of progesterone for traumatic brain injury (ProTECT III and SyNAPSE) have been disappointing (68). This is further evidence that the sex differences in brain injury and repair may not be based on purely hormonal difference.

#### Other Factors

In the face of HI injury, sex hormones may also play a role in hippocampal injury and later brain function. Waddell et al. using term-equivalent rat pups and the Vannucci model of HI, treated male, and females with estradiol immediately after the injury. Day 3 histological examination showed that drug therapy increased cell genesis in the female CA1, CA3, and dentate nucleus with no change in the male. Cell genesis was increased in these same areas in both sexes on day 7. However, on longer follow-up during behavioral testing, the HI males had significant deficits compared to the females who often exhibited no effect of HI or drug treatment. Drug treatment did seem to benefit the treated males vs. control HI animals. Testing included negative geotaxis, wire suspension, open field testing, novel object recognition, social play, and the Morris water-maze. In general males were negatively affected by HI on all tasks. Estradiol improved performance on some tasks but not all. HI did not negatively affect female rats in a number of tasks and estradiol did not appear to have an effect. In those tasks where females were negatively affected by HI, estradiol helped with wire suspension and social activity and not with the Morris water-maze (65).

#### Miscellaneous Differences in Males and Females

1. Lateralization: In the Rice-Vannucci model of hypoxic ischemic injury using 3-day old Wistar rats, it was shown that both sex and laterality affected injury and outcome. The outcome was worst in females with left sided brain injury (69). The sex difference may have been in part due to differences in the Na+/K+-ATPase responses (70). It has been shown in human peripheral blood lymphocytes that there is a sex difference in the expression and activity of this enzyme (71). In this study the investigators found differential expression of the Na+/K+ ATPase activity in the right and left side of the brain with the differential most pronounced in the female pups.
2. Adrenomedullin: The peptide adrenomedullin in the brain and CSF is protective against brain injury via its actions on vascular regulation of cerebral blood flow (72). It exerts its cellular actions through the ERK-MAPK (Extracellular signal-Related Kinase—Mitogen-Activated Protein Kinase) pathways of signal transduction in cells. The level of this peptide increases more significantly in female piglets than in males after percussive brain injury and appears to be neuroprotective in females. Adrenomedullin treatment or increasing ERK-MAPK activity in pig models of brain injury decreased brain injury and improved cerebral autoregulation selectively in males (72).
3. Nitric Oxide Synthase: Another modulator of injury and repair after hypoxic brain injury is the nitric oxide synthase system. It is not clear if there are sex dependent variations in activity of iNOS or nNOS *per se* but experiments in P7 rats using 2-Iminobiotin suggests that this compound is selectively neuroportective in females not through its NOS effects but probably via inhibition of initiation of cell death pathways at a level upstream of the activation of cytochrome c/caspase-3 dependent apoptosis (73).
4. G-protein coupled estrogen-receptor: The sex-hormonal mileu in the brain does not always correspond with estrogenic hormonal or receptor excess in females. Estrogen is known to exert its cellular effects through the estrogen α and β nuclear receptors. Recently a novel transmembrane cell membrane receptor termed G-protein-coupled estrogen receptor, GPER (aka GPR30) has been shown to be an important mediator of cell injury and repair in neural tissue and cerebral microvasculature and thus an important target for therapeutic intervention (74). GPER is widely distributed male and female brain but its levels rise more significantly after ischemic injury in males compared to females (both intact females and ovarectomized females) suggesting an non-sex hormone dependent effect (26, 75). Thus therapies using antagonists to the GPER may be more effective in males (26).

### Sex Differences Noted in Neonatal Clinical Studies

#### General Differences

There are some fundamental differences in placental and somatic growth in pregnancies with male and female fetuses. Female fetuses rely more on intrinsic placental growth while male fetuses are more dependent on transfer of nutrients across the placenta. Therefore, in times of maternal stress and deprivation, males are at higher risk of for growth retardation including brain growth (76, 77). Male fetuses are also more susceptible to head growth impairment. From data gathered after natural disasters and pregnancy outcomes in humans as in the case of Project Ice Storm in Canada, smaller head growth to birth length ratios was primarily seen in boys (78).

The basis for this observation may involve placental epigenetic changes involving the DNMT1 DNA methyltransferase enzyme and the differential effects of serotonin metabolism favoring increasing resilience in females compared to males (79). There is also evidence of modulation of fetal steroid exposure based on fetal sex which may partly be based on differential effects of the enzyme 11-β HSD (80). Moreover the effects of sex steroids may not be via cell death or proliferation but rather involve other components of neural functioning (65). There is suspicion however, that there may be other more fundamental cellular mechanisms that may be responsible for sex differences.

In a few retrospective studies it was shown that rates of IVH for similar gestational age premature VLBW infants were higher in males than in females (81, 82). Evidence from a large international series of neonatal ischemic stroke also showed a male predominance in the incidence of spontaneous and post-trauma arterial ischemic stroke suggesting the role of vascular factors (83). This points toward the above noted male dependence on placental nutrient/oxygen transfer capacity.

Recovery from respiratory distress syndrome was better in females not only for respiratory outcomes but also in cognitive scores on long term follow-up (84). A meta-analysis of clinical trials of preterm infants along with data from animal experiments reported from our center showed a cognitive advantage in females after hypoxic ischemic brain injury and the evidence was strong for overall and performance IQ measures (9, 85).

#### Differences in Medication Responses in Males and Females

Based on the demonstrated evidence of different anatomical, physiological, cellular, and molecular mechanisms in males and females, it is to be expected that the response to medications and treatments may also be different. Surprisingly, there has been very little recognition of this in clinical trials and in a majority of reports of outcomes following treatment, sex related differences are not reported. Below are some of the major neonatal clinical trials related to neuro protection.

1. Indomethacin: The importance of neonatal sex dimorphism in response to treatment strategies in infants is best illustrated by the studies on prophylactic indomethacin for prevention of intraventricular hemorrhage (IVH) in premature infants in the perinatal period. An initial RCT by Ment et al. showed that prophylactic indomethacin given to very low-birth-weight premature infants soon after birth helped in reducing the incidence and severity of IVH in the brain (13). This finding was followed by wide-spread use of prophylactic indomethacin in this population. By 36 and 54 month follow up of these infants difference in major developmental outcomes had disappeared (86, 87). Analysis of outcomes at 8 years of age in this cohort also showed no differences in neurodevelopmental outcome but on sub-group analysis male sex seemed to have more benefit from this therapy (88). When the original data were re-analyzed several years later entering male sex as a primary variable, it was discovered that only the male infants had benefited from prophylactic indomethacin (14). To test whether indomethacin prophylaxis had a sex-mediated effect on severe intraventricular hemorrhage (grade III and IV) and on long-term outcomes in extremely-low-birth-weight infants, a secondary analysis was performed in the entire “Trial of Indomethacin Prophylaxis in Preterms study” cohort. The results documented a differential treatment effect of indomethacin by sex with male infants showing more benefits of the therapy than females (89). Had there been sex based evaluation of outcomes in the initial study by Ment et al. the use of this therapy could have been more specifically applied.
2. Other therapies for neuroprotection in premature and newborn babies may have sexual dimorphisms and specific sex-related benefits and harms may have been overlooked.
3. Caffeine: Neonatal caffeine treatment (adenosine receptor antagonist, 15 mg/kg/day, between postnatal days 3 and 12) affects respiratory patterns in adult male but not female rats as shown by an increase in the respiratory frequency in the early phase of response to hypoxia and an increase in the tidal volume in the late phase of response. After treatment with neonatal caffeine (NCT) or water (NWT), they found that that NCT induces long-term changes in the adenosine receptor system. These changes may partially explain the modifications of the respiratory pattern induced by NCT in adults. The increased expression of the adenosine Adenosine(2A) receptor (specific to male rats), combined with the decreased tyrosine hydroxylase expression in the carotid body, suggests that NCT affects adenosine-dopamine interactions regulating chemosensory activity (90).
4. Chronic administration of an adenosine receptor antagonist disturbs spatial learning and memory in adult mice and neonatal caffeine exposure results in long-term behavioral and biochemical sequelae in mice and rats. But these changes varied based on the sex of the study animals (mice and rats). Neonatal caffeine exposure significantly improved retention in females (*P* <0.01) and significantly decreased retention in males (*P* <0.05). Thus, caffeine exposure limited to the first week of life resulted in alterations in passive avoidance retention that became apparent over pubertal development. These changes were a function of the sex of the animal (91).
5. Epidemiological studies indicate that caffeine consumption reduces the risk of Parkinson's disease (PD) in men, and antagonists of the adenosine 2A receptor ameliorate the motor symptoms of PD. These findings motivated Jones et al. to identify proteins whose expression is regulated by caffeine in a sexually dimorphic manner. After experimental manipulations in rat models, the researchers concluded that cytochrome oxidase is a metabolic target of caffeine and that stimulation of its activity by caffeine via blockade of A2AR signaling may be an important mechanism underlying the therapeutic benefits of caffeine in PD (92).
6. In the CAP trial of caffeine therapy in premature infants, which showed an improvement in respiratory parameters and chronic lung disease at discharge (93), there were also improvements in neurodevelopmental outcomes at 18–24 month follow up (94). However, there appeared to be no differences in neurodevelopmental outcomes noted at 5 years of follow-up (95). Since none of these studies were subjected to sex based analyses of data, any specific sex related benefits of therapy may have been masked. Given the accumulated evidence of sex-related brain injury and repair responses, a reappraisal of data is imperative before any further inferences are drawn. We are currently conducting studies to determine whether caffeine has greater benefits to male or female preterm equivalent rats who have undergone HI injury via the Rice Vannucci model (64).
7. Therapeutic Hypothermia: Another therapy for neonatal neuroprotection that has not been evaluated in a sex-specific manner is therapeutic hypothermia for hypoxic ischemic injury in the term neonate soon after birth (96–101). Studies conducted in North America, Europe and Australia have all shown benefits of therapeutic hypothermia whether used as whole-body cooling or by selective-head-cooling but in none of these studies were specific sex-based outcomes analyzed (102–108). Studies from Dietz et al. using different mice models are highly suggestive of sex-related differences in therapeutic hypothermia (109). In the mouse cardiac arrest/CPR model of brain injury, male, and female juvenile mice showed equivalent neuronal injury following CA/CPR; and hypothermia protects both sexes. However, there was a sexually dimorphic response to mild therapeutic hypothermia protection of synaptic function, where males needed a deeper level of hypothermia for equivalent synaptic protection. Studies from our lab using the Rice-Vannucci model for hypoxia-ischemia in newborn animals have shown sex dimorphism following injury as well-application of hypothermia in the preterm rat pup (8–11).
8. Erythropoietin(Epo): Epo is an emerging therapy for neuroprotection in neonates. Epo shows sex-specific differences in hippocampal gene expression after brain injury; and variable distribution after high-dose rEpo treatment, based on single-gene, and gene set analyses (110). Evidence from animal studies has shown that this drug has more benefits in females with focal cerebral ischemia compared to male animals with similar lesions (111). There is currently a phase III trial of hypothermia and Epo for infants with moderate to severe HIE. Upon completion, this study has the opportunity to demonstrate if there is an added benefit of Epo to hypothermia and whether that benefit is greater in one sex or the other. There have been small clinical reports of Epo for HIE in human term infants. None of these studies have reported outcomes based on sex of the infant (112–117). Given the findings of sexual dimorphism from animal studies it is imperative that any analysis of clinical data includes sex-related differences in outcomes.
9. Prenatal maternal magnesium sulfate: Multiple studies have examined the effect of maternal administration of magnesium sulfate prior to an anticipated preterm birth (118–123). Virtually all of these clinical studies have found a decrease in cerebral palsy (CP) and mortality but no effect on cognitive function. However, none of the clinical studies have taken into account sex dimorphism (118, 124–127). Studies in term equivalent newborn piglets have shown that pre-HI administration of magnesium interacts with the NMDA post synaptic receptor in the cortex to prevent influx and release of protein bound calcium (128). However, animal studies designed to examine the mechanism for the prevention of CP in preterm infants have failed to determine mechanism (129, 130).

## Conclusions

There are clearly differences in brain structure, response to sex hormones, hypoxic ischemic injury, and responses to interventions for HI injury in male and female fetuses, or newborn preterm and term babies. The mechanisms for the differences continue to be uncovered. However, it is most important for clinicians to be aware of these sex based differences in provision of acute care and long term intervention in order for these children to have optimal neurologic outcomes.

## Author Contributions

TR: primary author who generated topic, did majority of writing and editing, and contributed original research. ZH: did original research for paper, organized preliminary manuscript. RF: senior investigator for Fitch Lab, contributed original research and editing of manuscript.

### Conflict of Interest Statement

The authors declare that the research was conducted in the absence of any commercial or financial relationships that could be construed as a potential conflict of interest.
